# The IL-6R and Bmi-1 axis controls self-renewal and chemoresistance of head and neck cancer stem cells

**DOI:** 10.1038/s41419-021-04268-5

**Published:** 2021-10-23

**Authors:** Alexandra E. Herzog, Kristy A. Warner, Zhaocheng Zhang, Emily Bellile, Meera A. Bhagat, Rogerio M. Castilho, Gregory T. Wolf, Peter J. Polverini, Alexander T. Pearson, Jacques E. Nör

**Affiliations:** 1 Department of Cariology, Restorative Sciences, Endodontics, University of Michigan School of Dentistry, Ann Arbor, MI USA; 2 Department of Biostatistics, University of Michigan School of Public Health, Ann Arbor, MI USA; 3 Department of Periodontics and Oral Medicine, University of Michigan School of Dentistry, Ann Arbor, MI USA; 4 Department of Otolaryngology-Head and Neck Surgery, University of Michigan Medical School, Ann Arbor, MI USA; 5 Department of Pathology, University of Michigan Medical School, Ann Arbor, MI USA; 6 Department of Biomedical Engineering, University of Michigan College of Engineering, Ann Arbor, MI USA; 7 Department of Hematology/Oncology, University of Chicago Medicine and Biological Sciences, Chicago, IL USA; 8 University of Michigan Rogel Cancer Center, Ann Arbor, MI USA

**Keywords:** Cancer stem cells, Cell death, Self-renewal

## Abstract

Despite major progress in elucidating the pathobiology of head and neck squamous cell carcinoma (HNSCC), the high frequency of disease relapse correlates with unacceptably deficient patient survival. We previously showed that cancer stem-like cells (CSCs) drive tumorigenesis and progression of HNSCC. Although CSCs constitute only 2–5% of total tumor cells, CSCs contribute to tumor progression by virtue of their high tumorigenic potential and their resistance to chemo-, radio-, and immunotherapy. Not only are CSCs resistant to therapy, but cytotoxic agents actually enhance cancer stemness by activating transcription of pluripotency factors and by inducing expression of Bmi-1, a master regulator of stem cell self-renewal. We hypothesized therapeutic inhibition of interleukin-6 receptor (IL-6R) suppresses Bmi-1 to overcome intrinsic chemoresistance of CSCs. We observed that high Bmi-1 expression correlates with decreased (*p* = 0.04) recurrence-free survival time in HNSCC patients (*n* = 216). Blockade of IL-6R by lentiviral knockdown or pharmacologic inhibition with a humanized monoclonal antibody (Tocilizumab) is sufficient to inhibit Bmi-1 expression, secondary sphere formation, and to decrease the CSC fraction even in Cisplatin-resistant HNSCC cells. IL-6R inhibition with Tocilizumab abrogates Cisplatin-mediated increase in CSC fraction and induction of Bmi-1 in patient-derived xenograft (PDX) models of HNSCC. Notably, Tocilizumab inhibits Bmi-1 and suppresses growth of xenograft tumors generated with Cisplatin-resistant HNSCC cells. Altogether, these studies demonstrate that therapeutic blockade of IL-6R suppresses Bmi-1 function and inhibits cancer stemness. These results suggest therapeutic inhibition of IL-6R might be a viable strategy to overcome the CSC-mediated chemoresistance typically observed in HNSCC patients.

## Introduction

Head and neck squamous cell carcinoma (HNSCC) is the sixth most common solid tumor experiencing around 55,000 newly diagnosed cases every year in the United States [1]. Treatment modalities for advanced HNSCC include surgical resection, radiation and chemotherapy, or chemotherapy alone, which commonly correlate with increased patient morbidity and disease relapse [2]. Platinum-based agents persist as standard of care in chemotherapeutic treatment of HNSCC, of which Cisplatin is the most commonly used. Despite their well-recognized survival benefit through refined control of tumor growth, treatment with platinum-based chemotherapeutics is frequently associated with the development of evasive resistance leading to tumor progression [3]. In HNSCC, locoregional recurrence accounts for 20–40% of the 5-year patient mortality rate [4], making it imperative that a treatment strategy that is more consistently effective be investigated. This is particularly true in human papillomavirus (HPV)-negative HNSCC patients, as HPV-negative disease exhibits worse prognosis and higher recurrence rates when compared to HPV-positive disease [5]. Improved comprehension of the pathobiology of HNSCC will enable establishing novel mechanism-based strategies to ameliorate the survival and health standard of HNSCC patients.

The cancer stem cell hypothesis proposes that key malignant features of neoplastic cells originate from acquisition of stem-like features [6]. Cancer stem-like cells (CSCs) encompass a unique cellular subpopulation characterized by multipotency, uniquely high tumorigenic potential, and self-renewal. CSCs in HNSCC are identified by high activity of aldehyde dehydrogenase (ALDH) and high expression of the surface glycoprotein CD44 [7–10]. These cells drive tumor initiation, tumor progression, and, ultimately, therapeutic evasion in HNSCC [7, 11, 12]. Thus, targeted therapeutic ablation of CSCs might benefit head and neck cancer patients.

Traditional cytotoxic chemotherapy is known to instigate phenotypic changes in cancer cells [13] by causing a shift towards self-renewal in the tumorigenic CSC population, priming a more aggressive phenotype in residual tumor cells that leads to tumor recurrence or metastatic spread [14, 15]. Cisplatin increases the head and neck CSC fraction and induces expression of Bmi-1, a master regulator of stem cell self-renewal [16]. Clinical observations suggest that chemoresistant tumor cells possess the capacity to initiate a new tumor, resulting in either locoregional recurrence or metastasis [17]. It has been recently demonstrated that Bmi-1^+^ cancer stem cells mediate chemoresistance and metastasis in HNSCC [18]. In HNSCC, Cisplatin-resistant cancer cells display a distinct increase in expression of Bmi-1 among other stemness markers, as opposed to Cisplatin-sensitive cancer cells [13, 16]. Recent work evaluating immunotherapy resistance in HNSCC have also found CSC to be a relevant immune-oncology target [19–21].

The molecular crosstalk within the tumor microenvironment has been shown to assume a crucial part in maintaining the CSC pool and mediating HNSCC resistance to conventional chemotherapy [22, 23]. It has been previously shown that interleukin-6 (IL-6) secreted from endothelial cells within the perivascular niche enhances CSC survival, self-renewal, and tumorigenic potential [24, 25]. Cisplatin exposure activates the IL-6 pathway [26], which potentiates the Cisplatin induction of the CSC fraction and Bmi-1 expression [16]. We have shown that head and neck CSCs (ALDH^high^CD44^high^) exhibit higher levels of IL-6 receptor (IL-6R) expression when compared with non-CSC, i.e., ALDH^low^CD44^low^ [24, 25]. We have also reported that inhibition of IL-6R signaling is sufficient to decrease the fraction of head and neck CSCs [24, 25]. Notably, high levels of tumor IL-6R and serum IL-6 expression are strongly correlated with poor survival of patients with HNSCC [25, 27].

Here we used Tocilizumab (Genentech), a humanized monoclonal anti-IL-6R antibody approved by the US Food and Drug Administration (FDA) since 2010 for rheumatoid arthritis as a prototypic inhibitor of the IL-6R signaling pathway. We demonstrated that therapeutic blockade of IL-6R inhibits Bmi-1 function and suppresses Cisplatin-induced CSC self-renewal and tumor growth. In summary, these data suggest that therapeutic inhibition of IL-6R might be a viable strategy to overcome CSC-mediated chemoresistance in head and neck cancer.

## Results

### IL-6/Bmi-1 signaling axis regulates cancer cell self-renewal and correlates with recurrence-free survival of HNSCC patients

**Fig. 1:**
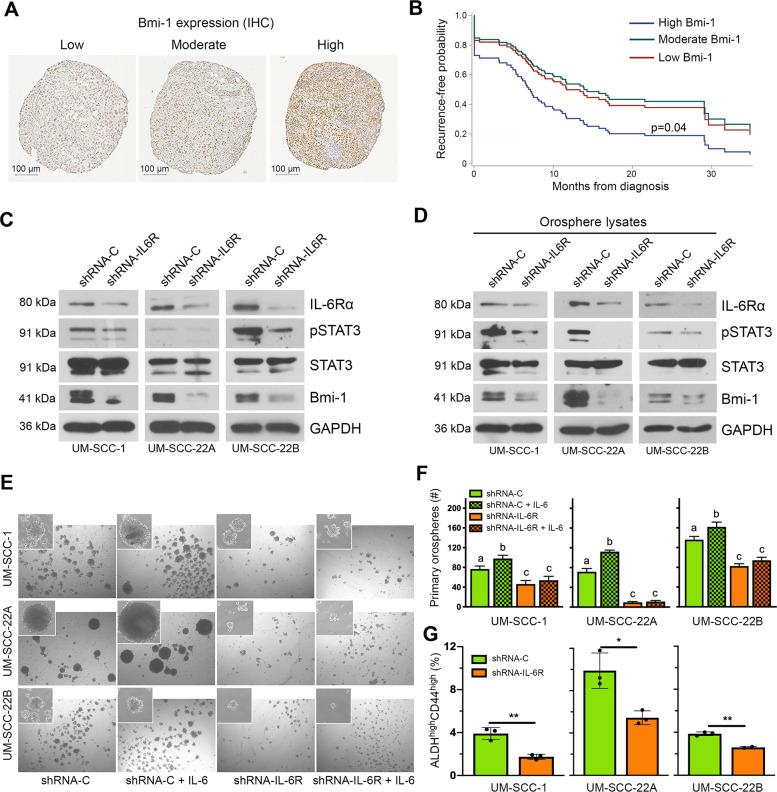
IL-6/Bmi-1 signaling axis regulates cancer cell self-renewal and correlates with recurrence-free survival of HNSCC patients. **A** Immunohistochemistry staining for Bmi-1 in human HNSCC tumor cores of a tissue microarray. Representative images of staining patterns in Bmi-1-low, Bmi-1-moderate, and Bmi-1-high specimens. **B** Graph depicting adjusted recurrence-free survival function over time in tumors with Bmi-1 expression separated into intensity tertiles. **C** Western blottings showing baseline protein levels (IL-6R, phosphorylated STAT3, total STAT3, and Bmi-1) in HNSCC cells (UM-SCC-1, -22A, and -22B) stably transduced with lentiviral vectors expressing shRNA-IL-6R or scramble sequence control (shRNA-C). **D** Western blottings showing protein levels in lysates prepared from primary orospheres generated by HNSCC cells stably transduced with lentiviral vectors expressing shRNA-IL-6R or shRNA-C. **E** Representative images (×40) of primary orospheres (day 8) formed by IL-6R-silenced or vector control cells. Cells were treated with 20 ng/ml rhIL-6 the day after plating in ultra-low attachment (ULA) orosphere conditions. Inserts at ×100 magnification. **F** Graph depicting the number of primary orospheres per well. Bar graphs display mean ± SD from five fields per well in three wells per condition. **G** Flow cytometry graphs depicting the CSC fraction (ALDH^high^CD44^high^ cells) in IL-6R knockdown and control cells. Bar graphs display mean ± SD (*n* = 3). *p < 0.05 or **p < 0.01 as determined by *t*-test. Different lowercase letters indicate statistical significance at *p* < 0.05.

To perceive the clinical significance of Bmi-1 function in HNSCC patients, a tissue microarray (TMA) of human HNSCC tumors (*n* = 216) was independently evaluated for Bmi-1 staining by two trained oral pathologists blind for patient outcome. Immunostaining for Bmi-1 was almost exclusively nuclear, varying from mild to intense and primarily associated with nuclear chromatin, resulting in a granular-like pattern (Fig. 1A). Bmi-1 expression formed a gradient towards high intensity in the basal epithelial layer, where stem cells reside in normal oral epithelium. Bmi-1 expression clearly correlated with shorter recurrence-free survival (*p* = 0.04) (Fig. 1B). No association was found between Bmi-1 expression and gender (*p* = 0.30), age (*p* = 0.82), tobacco use (*p* = 0.96), or clinical stage (*p* = 0.92), propounding that Bmi-1 may cogitate an impartial identifier of tumor recurrence. High levels of pretreatment tumor IL-6R and serum IL-6 expression have been shown to correlate with a higher rate of tumor recurrence and reduced survival of HNSCC patients, which emphasizes the relevance of inhibiting this signaling pathway to mitigate the risk of recurrence in HNSCC [25, 27]. To assess the role of the IL-6R signaling pathway in maintaining the CSC population and in self-renewal, we used lentiviral short hairpin RNA (shRNA) constructs to knock down IL-6R expression in the UM-SCC-1, UM-SCC-22A, and UM-SCC-22B cells. Western blottings revealed that IL-6R silencing was sufficient to inhibit STAT3 phosphorylation and Bmi-1 expression (Fig. 1C). Notably, we have previously shown that CSCs exhibit constitutive phosphorylation of STAT3 and high expression of Bmi-1 [24]. To determine whether IL-6R knockdown affects expression of these signaling factors in CSCs, we used the orosphere assay to functionally enrich cell cultures for CSCs [28]. We observed that IL-6R silencing also inhibited STAT3 phosphorylation and Bmi-1 expression in CSC-enriched orospheres (Fig. 1D). Moreover, IL-6R silencing decreased the orosphere-forming ability of all HNSCC cell lines evaluated by reducing both the number and the size of spheres, as compared to cells transduced with shRNA-control constructs (Fig. 1E, F and Supplementary Fig. S1). Lastly, to determine the effect of IL-6R silencing on the CSC fraction directly, flow cytometry analysis showed a decrease in ALDH^high^CD44^high^ cells in these cells when compared to vector controls (Fig. 1G and Supplementary Fig. S1). Collectively, these data underline the significance of IL-6R signaling in maintaining the stemness phenotype and self-renewal of head and neck CSCs.

### Therapeutic inhibition of IL-6R abrogates Cisplatin-induced cancer stemness in vivo

**Fig. 2:**
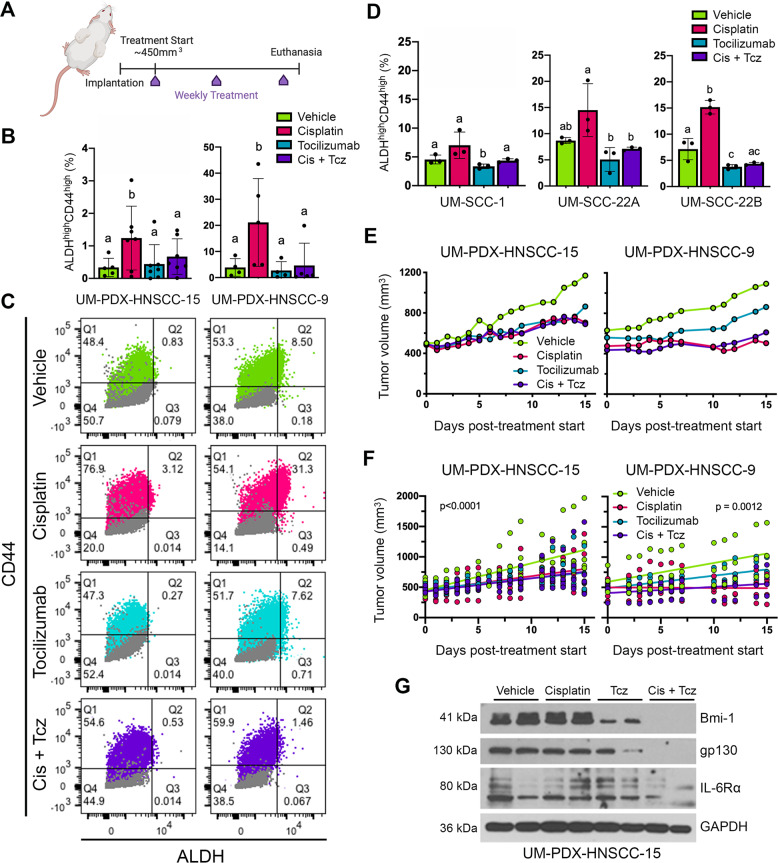
Tocilizumab suppresses Cisplatin-induced stemness in PDX models of HNSCC in vivo. **A** Schematic drawing depicting study design. Mice harboring PDX tumors began weekly treatment for 2 weeks (3 doses total), receiving either no treatment, Cisplatin (5 mg/kg, i.p.), and/or Tocilizumab (10 mg/kg, i.p.). Mice were killed 2 days after last dose. **B** Bar graphs depicting percentage of CSCs (ALDH^high^CD44^high^ cells) in PDX tumors, as measured by flow cytometry. Different lowercase letters indicate statistical significance at *p* < 0.05. **C** Representative flow cytometry charts depicting DEAB/IgG controls (gray) for Aldefluor (ALDH activity) and CD44 expression. One experimental replicate per group is shown to demonstrate gate-setting strategy. ALDH^high^CD44^high^ cells were identified based on these gates. **D** Bar graphs depicting the percentage CSCs (ALDH^high^CD44^high^ cells) in HNSCC cell lines, as determined by flow cytometry. Bar graphs display mean ± SD (*n* = 3). Different lowercase letters indicate statistical significance at *p* < 0.05. **E** Line graph depicting mean tumor volume over time in the PDX models after treatment with Cisplatin and/or Tocilizumab. Tumor measurements were taken three times per week until study endpoints. **F** Simple linear regression model of mean tumor volumes over the duration of the experiment. **G** Western blottings of representative PDX tumor tissue lysates from each treatment group.

Conforming to the cancer stem cell hypothesis, therapeutic eradication of CSCs prevents tumor progression and therapeutic resistance [6]. As shown above and in previous publications [24, 25], the IL-6 pathway is a particularly attractive target for CSC-specific therapy. To determine the outcome of combination therapy with Cisplatin and IL-6R inhibitor Tocilizumab on the CSC fraction, we utilized HNSCC patient-derived xenograft (PDX) mouse models characterized by our laboratory [29]. When tumors grew to an average of ~450 mm^3^ (Supplementary Fig. S2A–C), we began weekly treatment with Cisplatin and/or Tocilizumab for 2 weeks (Fig. 2A).

To evaluate the consequence of this treatment on the CSC fraction of PDX tumors in vivo, we conducted flow cytometry for ALDH activity and CD44 expression (Fig. 2B, C). Consistent with previously published findings [16], we observed here that Cisplatin is sufficient to increase the CSC fraction in HNSCC (Fig. 2B). Importantly, Tocilizumab decreased the CSC fraction and abrogated Cisplatin induction of CSC fraction in both PDX models. These results were corroborated in vitro, as Tocilizumab abrogated Cisplatin-induced increase of the CSC fraction in UM-SCC-1, -22A, and -22B cell lines in vitro (Fig. 2D). Although the principal objective of this short-term experiment was to determine the treatment effect on tumor CSC fraction, we also observed significant suppression of tumor growth by Cisplatin and combination therapy, as compared to the untreated group (Fig. 2E, F). Tocilizumab alone suppressed tumor growth only in the UM-PDX-HNSCC-15 model. Notably, these data illustrate that although Cisplatin alone is effective in slowing down tumor growth, a combination therapy approach with Tocilizumab is required to decrease tumor CSC fraction (Fig. 2A). Likewise, a CSC-targeted therapy only targets a relatively small fraction of cancer cells and, therefore, may not inhibit tumor growth alone. In a parallel study, we assessed the effect of this treatment strategy on long-term tumor regrowth using a scaffold xenograft model with UM-SCC-22B cells. In this experiment, Cisplatin therapy was halted after 2 weeks, whereas maintenance injections of Tocilizumab were continuously administered weekly (Supplementary Fig. S2A). We observed a delay in tumor regrowth in the groups that received Tocilizumab (Supplementary Fig. S2D, E). Western blottings of representative PDX tumor lysates showed that Tocilizumab inhibited Bmi-1 expression in vivo, even in the presence of Cisplatin (Fig. 2G). These findings support the flow cytometry data and demonstrate that Tocilizumab inhibits Cisplatin-induced cancer stemness.

### Tocilizumab suppresses Cisplatin induction of CSC-associated signaling pathways

**Fig. 3:**
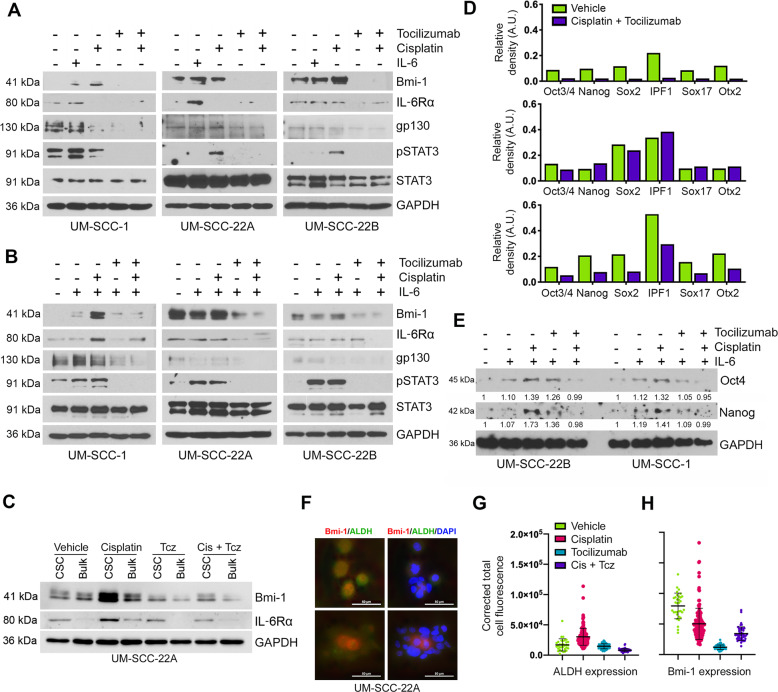
IL-6R blockade with Tocilizumab suppresses Cisplatin-induced stemness in vitro. **A** Western blot analyses of Bmi-1, IL-6R, gp130, pSTAT3, and STAT3 in UM-SCC-1, UM-SCC-22A, and UM-SCC-22B cells treated with vehicle, rhIL-6 (20 ng/ml), Cisplatin (1 µM), and/or Tocilizumab (0.1 µM). **B** Western blot analysis of UM-SCC-1, UM-SCC-22A, and UM-SCC-22B cells treated with rhIL-6 (20 ng/ml) combined with vehicle, Cisplatin (1 µM), and/or Tocilizumab (0.1 µM) for 24 h, followed by additional rhIL-6 (20 ng/ml) for 24 h. **C** Western blottings to evaluate the effect of 1 µM Cisplatin and/or 0.1 µM Tocilizumab on UM-SCC-22A cells sorted for ALDH/CD44. **D** Stem cell marker protein array analysis of UM-SCC-1, UM-SCC-22A, and UM-SCC-22B cells treated with Cisplatin (1 µM) and Tocilizumab (0.1 µM), as compared to vehicle controls. **E** Western blot analysis of stem cell markers (Oct4 and Nanog) in UM-SCC-1 and UM-SCC-22B cells treated with rhIL-6 (20 ng/ml), Cisplatin (1 µM), and/or Tocilizumab (0.1 µM). **F** Representative images of immunofluorescence staining (Bmi-1 and ALDH1) of UM-SCC-22A cells plated in chamber slides and treated with Cisplatin (0–1 µM) and/or Tocilizumab (0–0.1 µM). **G** Graph quantifying mean cellular fluorescence of ALDH1 expression normalized to DAPI in UM-SCC-22A cells treated with Cisplatin (1 µm) and/or Tocilizumab (0.1 µM). **H** Graph quantifying mean cellular fluorescence of Bmi-1 normalized to DAPI stain in UM-SCC-22A cells treated with Cisplatin (1 µM) and/or Tocilizumab (0.1 µM).

To begin elucidating the mechanisms underlying the anti-CSC effect of therapeutic blockade of IL-6R, we examined the impact of the IL-6 pathway on downstream signaling effectors in three HNSCC cell lines. Cells were exposed to Tocilizumab and/or Cisplatin (Fig. 3A). In a parallel set of experiments, we exposed cells to Cisplatin and/or Tocilizumab in the presence of rhIL-6 (Fig. 3B). We observed that Cisplatin activated STAT3 and induced Bmi-1 expression (Fig. 3A). Tocilizumab inhibited the expression of IL-6R and gp130, STAT3 activation, and Bmi-1 expression, even in the presence of excess rhIL-6 and/or Cisplatin (Fig. 3A, B). To determine the effect of Tocilizumab in CSCs vs. bulk tumor cells, we sorted cells for ALDH/CD44 and performed western blot analyses. We observed that Cisplatin induces expression of Bmi-1 expression in sorted CSCs, and that Tocilizumab abrogates Cisplatin-induced Bmi-1 in CSCs (Fig. 3C).

To determine whether the anti-stemness effect of Tocilizumab was specific to Bmi-1 or also affected other stemness pathways, we used a stem cell protein array. Combination therapy with Tocilizumab/Cisplatin decreased the expression of several pluripotent stem cell markers as compared to controls (Fig. 3D and Supplementary Fig. S3B), suggesting that this therapeutic strategy may broadly suppress stemness phenotypes in HNSCC. To verify these results, we analyzed protein expression of Oct4 and Nanog via western blotting, as these traditional stem cell markers have been implied in the maintenance of head and neck CSC [30, 31]. Interestingly, although IL-6 and Cisplatin both induced expression of Oct4 and Nanog, Tocilizumab suppressed more effectively the expression of these two regulators of stemness when used in combination with Cisplatin than when Tocilizumab was used alone (Fig. 3E).

To determine whether the change in cancer stem cell fraction occurs due to a numerator or a denominator effect, we used immunocytochemistry to evaluate ALDH1 and Bmi-1 expression on a single-cell basis (Fig. 3F and Supplementary Fig. S3A). Interestingly, Cisplatin induced an overall shift in ALDH1 fluorescence, with a small subpopulation of cells expressing very high levels of ALDH1 (Fig. 3G). Cisplatin did not appear to induce an overall shift in Bmi-1 fluorescence but also sharply increased its expression in a small proportion of cells (Fig. 3H). Tocilizumab and the combination therapy resulted in an overall suppression of ALDH1 and Bmi-1. Overall, these data suggest that IL-6R signaling regulates HNSCC stemness, and that combination therapy with Cisplatin and Tocilizumab prevents acquisition of the stem-like phenotype within HNSCC cells observed upon single-agent Cisplatin exposure.

### Tocilizumab inhibits STAT3 signaling and self-renewal of HNSCC cells

**Fig. 4:**
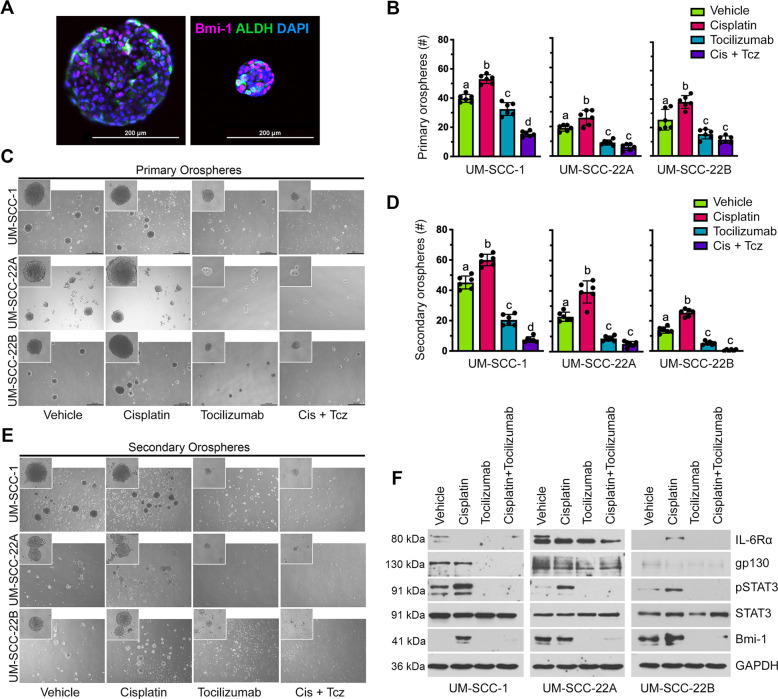
Tocilizumab prevents Cisplatin-induced self-renewal of cells in orospheres. **A** Representative images of immunofluorescence staining of untreated, cryosectioned orospheres after 8 days in suspension culture. Example images of both a large (left) and small (right) orosphere are shown. **B** Bar graphs depicting the mean number ± SD of primary orospheres per well (*n* = 6 per treatment group). Different lowercase letters indicate statistical significance at *p* < 0.05. **C** Representative images (×40) of primary orospheres 8 days after treatment with Cisplatin (1 µm) and/or Tocilizumab (0.1 µm). Cells were treated the day after seeding in ultra-low attachment plates. Inserts at ×100 magnification. **D** Bar graphs depicting the mean number ± SD of secondary orospheres per well (*n* = 6 per treatment group). Different lowercase letters indicate statistical significance at *p* < 0.05. **E** Representative images (×40) of secondary orospheres on day 8 after treatment with Cisplatin (1 µM) and/or Tocilizumab (0.1 µM). Cells were treated the day after seeding in ultra-low attachment plates. Inserts at ×100 magnification. **F** Western blottings showing protein levels Bmi-1, IL-6R, gp130, pSTAT3, and STAT3 in lysates prepared from primary orospheres on day 8 after treatment with Cisplatin (1 µM) and/or Tocilizumab (0.1 µM).

To evaluate the consequence of Tocilizumab on inhibiting CSC stemness and self-renewal, we engaged the orosphere assay. Utilizing the orosphere assay enables functional measurement of stemness and self-renewal of CSCs in ultra-low attachment (ULA) conditions [28]. Although primary orospheres serve as a read-out of cancer cell stemness, serial passaging of these cultures into secondary orospheres allows assessment of their self-renewal ability. We also sought to understand Bmi-1 and ALDH expression patterns within orospheres. It is known that CSC initiate orosphere formation, and that orospheres express higher levels of ALDH, IL-6R, and Bmi-1 than cells under standard attachment conditions [28, 32, 33]. We found that within both small and large spheres, only a subset of cells expresses high levels of ALDH and Bmi-1. Interestingly, not all ALDH-expressing cells also express Bmi-1 and vice versa, which may suggest a self-renewing subpopulation of CSC (Fig. 4A). These findings mimic the heterogeneity of ALDH and Bmi-1 expression within a tumor.

Cisplatin treatment increased the number (Fig. 4B) and size (Fig. 4C) of primary orospheres, as well as enhanced their growth over time (Supplementary Fig. S4A, B), which is consistent with Cisplatin-induced increase in the CSC fraction (Fig. 2A, B). In contrast, treatment with Tocilizumab suppressed orosphere formation, significantly decreasing both size and number of orospheres (Fig. 4B, C). The combination therapy had a similar effect to Tocilizumab alone but was more effective in decreasing sphere number formed by UM-SCC-1 cells. Tocilizumab alone or in combination with Cisplatin also suppressed the Cisplatin induction of the number and size of secondary orospheres (Fig. 4D, E). Although Cisplatin alone did not further increase the size of secondary orospheres (Supplementary Fig. S4C), combination therapy nearly eliminated secondary orosphere formation (Fig. 4D). Notably, Tocilizumab suppresses the dose-dependent induction of Bmi-1 by Cisplatin (Supplementary Fig. S4D–F).

To validate our findings of protein expression changes presented in Fig. 3A, B, we next sought to determine whether Tocilizumab inhibits STAT3 activation and Bmi-1 expression in the orospheres. Orosphere protein lysates showed via western blot analysis that Cisplatin induces STAT3 activation within the spheres, and that Tocilizumab suppresses this activation even in the presence of Cisplatin (Fig. 4F). As in Fig. 3A, B, Tocilizumab decreased the expression of both IL-6 co-receptors. Interestingly, Cisplatin further induced Bmi-1 expression within orospheres from UM-SCC-1 cells but did not further increase Bmi-1 within UM-SCC-22A and UM-SCC-22B spheres. Tocilizumab fully suppressed Bmi-1 expression in orospheres, even in the presence of Cisplatin (Fig. 4F). These findings further support IL-6 signaling as a pivotal regulator of cancer stem cell self-renewal and overcoming Cisplatin induction of CSC function.

### Therapeutic inhibition of IL-6R decreases self-renewal and CSC fraction in Cisplatin-resistant HNSCC cells

**Fig. 5:**
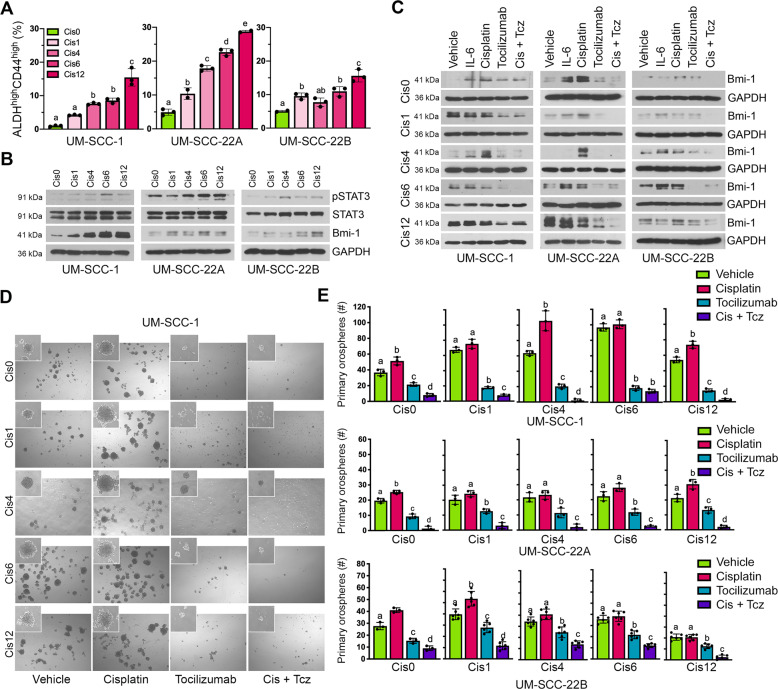
Tocilizumab decreases the CSC fraction and the self-renewal of Cisplatin-resistant HNSCC cells. **A** Bar graphs depicting the CSC fraction (ALDH^high^CD44^high^ cells) in Cisplatin-naive and Cisplatin-resistant variants of UM-SCC-1, UM-SCC-22A, and UM-SCC-22B cells, as determined by flow cytometry. Graphs display mean ± SD (*n* = 3) and significance denoted by different lowercase letters at *p* < 0.05. **B** Western blottings showing baseline expression of phosphorylated STAT3, total STAT3, and Bmi-1 expression in Cisplatin-naive and Cisplatin-resistant HNSCC cell line variants. **C** Western blot analysis of Bmi-1 expression after treatment with rhIL-6 (20 ng/ml), Cisplatin (1 µm), and/or Tocilizumab (0.1 µM). **D** Representative images (×40) of Cisplatin-naive and Cisplatin-resistant UM-SCC-1 primary orospheres on day 8 after treatment with Cisplatin (1 µM) and/or Tocilizumab (0.1 µM). Cells were treated the day after seeding in ultra-low attachment plates. Inserts at ×100 magnification. **E** Bar graphs depicting number (mean ± SD) of primary orospheres per well, from three wells per experimental condition. Different lowercase letters indicate statistical significance at *p* < 0.05.

Clinical and laboratory observations suggest a subset of tumor cells are chemoresistant and acquire a migratory behavior, ultimately giving rise to the process of evasive resistance [34]. CSC cells are known to be critical mediators of therapeutic evasion and resistance [6, 7, 12], suggesting that targeted elimination of this cellular subpopulation is necessary to prevent disease recurrence. To determine the efficacy of IL-6R inhibition as an avenue to conquer Cisplatin resistance, we used Cisplatin-resistant variants of the UM-SCC-1, -22A, and -22B cell lines that were generated in our lab [16]. These variants were named Cis1, Cis4, Cis6, Cis12, each being resistant to the corresponding concentration of Cisplatin (µM). The naive parent cells are here referred to as Cis0. First, we examined the CSC fraction in the Cisplatin-resistant variants as compared to the naïve parent cells (Fig. 5A and Supplementary Fig. S5A). The Cisplatin-resistant HNSCC cells showed a dose-dependent increase in the ALDH^high^CD44^high^ CSC fraction (Fig. 5A) as compared to the parent cell lines, likewise activation of STAT3 signaling and expression of Bmi-1 (Fig. 5B). This observation supports previously published findings of increases in CSC fraction and stemness markers in Cisplatin-resistant cells [13, 16]. Next, we evaluated whether IL-6R inhibition with Tocilizumab could decrease Bmi-1 expression of Cisplatin-resistant cells. Cells were treated as in previous experiments with either rhIL-6, Cisplatin, and with and without Tocilizumab. We observed that in all three Cisplatin-resistant HNSCC cell line sets, as well as each corresponding resistant variant, Tocilizumab suppressed Bmi-1 expression, even in the presence of Cisplatin treatment (Fig. 5C). Interestingly, treatment with Cisplatin further induced expression of Bmi-1 as compared to untreated cell line variants with lower Cisplatin resistance. However, Cisplatin did not further enhance Bmi-1 expression in the Cis12 cell lines, potentially indicating a saturated level of resistance (Fig. 5C). To address the efficacy of Tocilizumab in the inhibition of CSC self-renewal, the orosphere assay was employed (Fig. 5D and Supplementary Fig. S6A). Cisplatin increased orosphere formation in naive HNSCC cells. In the Cisplatin-resistant variants, Cisplatin treatment either increased or did not further induce sphere-forming ability. However, Tocilizumab reduced the number and size of orospheres in all cell lines, both alone and in combination with Cisplatin (Fig. 5E and Supplementary Fig. S6B). Of note, the combination therapy was found to have a synergistic effect on decreasing sphere formation in essentially all cell lines evaluated here, including the Cisplatin-resistant cells (Supplementary Fig. S6C).

### Tocilizumab suppresses growth and Bmi-1 expression of Cisplatin-resistant xenografts

**Fig. 6:**
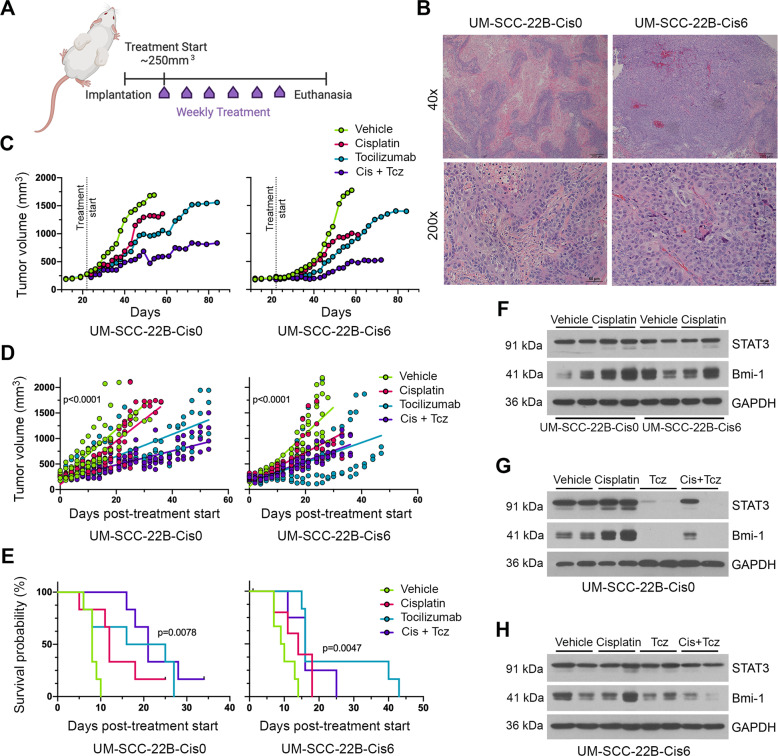
Tocilizumab inhibits cancer stemness in a Cisplatin-resistant xenograft model of HNSCC. **A** Schematic diagram of the study design. Xenograft tumors were generated with UM-SCC-22B-Cis0 (Cisplatin-naive) or UM-SCC-22BCis6 (Cisplatin-resistant) cells. Treatment began once tumors reached an average of 250 mm^3^. Mice received weekly injections of either vehicle, Cisplatin (5 mg/kg, i.p.) and/or Tocilizumab (10 mg/kg, i.p.) for up to 8 weeks. Mice were killed either at experiment endpoint (8 weeks post treatment start) or when reaching our cutoff tumor volume (2000 mm^3^). **B** Representative images of histological sections stained for H&E of xenograft tumors generated with UM-SCC-22B-Cis0 or UM-SCC-22BCis6 cells. Scale bars represent 200 µm at ×40 magnification and 50 µm at ×200 magnification. **C** Line graph depicting mean tumor volume over time after treatment with vehicle, Cisplatin, and/or Tocilizumab. Tumor measurements were taken three times per week until study endpoints. **D** Simple linear regression model of mean tumor volumes over the duration of the experiment. **E** Kaplan–Meier graph for survival, as defined by time to doubling of tumor volume, as compared to pretreatment tumor volume (*n* = 6 per experimental condition). **F** Western blottings for STAT3 and Bmi-1 expression in representative xenograft tumor tissue lysates (UM-SCC-22B-Cis0 or UM-SCC-22BCis6 xenograft models) comparing vehicle-treated with Cisplatin-treated mice. **G** Western blottings for STAT3 and Bmi-1 in representative UM-SCC-22B-Cis0 (Cisplatin-naive) tumor lysates collected from mice treated with vehicle, Tocilizumab, and/or Cisplatin. **H** Western blottings for STAT3 and Bmi-1 in representative UM-SCC-22BCis6 (Cisplatin-resistant) tumor lysates collected from mice treated with vehicle, Tocilizumab, and/or Cisplatin.

To examine whether in vitro findings translate into in vivo results, we seeded UM-SCC-22B-naive and UM-SCC-22BCis6-resistant cells in biodegradable scaffolds embedded in SCID mice. We chose the UM-SCC-22BCis6 variant, because these cells had the highest level of Cisplatin resistance, while not exhibiting inhibition of cell proliferation (data not shown). When tumors reached an average of 250 mm^3^ (Supplementary Fig. S7G), we began weekly treatment with Cisplatin and/or Tocilizumab for up to 8 weeks (Fig. 6A). Histological analyses suggested that UM-SCC-22BCis6-resistant tumors were histologically less differentiated and more vascularized, when compared to UM-SCC-22B-naive tumors (Fig. 6B).

Treatment with Tocilizumab with or without Cisplatin slowed down tumor growth in both UM-SCC-22B and UM-SCC-22BCis6 xenograft tumors (Fig. 6C, D) and extended time to tumor volume doubling (Fig. 6E). In the resistant tumor model, Cisplatin was less effective in inhibiting tumor growth (Fig. 6C, D). In UM-SCC-22B-naive tumors, Cisplatin suppressed tumor growth more potently during earlier doses and was later observed to lose efficacy in inhibiting tumor growth. The combination therapy approach was most effective in both xenograft models. Western blotting analyses of tumor tissue lysates showed that Cisplatin induced Bmi-1 expression in UM-SCC-22B-naive tumors (Fig. 6F). After treatment with Tocilizumab, both STAT3 and Bmi-1 expression were inhibited in UM-SCC-22B tumors (Fig. 6G and Supplementary Fig. S7A–F). These data support our findings from our experiments using PDX models shown above (Fig. 2F). Notably, Tocilizumab also inhibited Bmi-1 expression in the Cis6-resistant variants, with or without concurrent Cisplatin treatment (Fig. 6H). However, Tocilizumab did not affect total STAT3 expression in these tumors. These data showed that therapeutic IL-6R inhibition with Tocilizumab might be an effective strategy to inhibit self-renewal and overcome Cisplatin resistance in HNSCC.

## Discussion

Platinum-based chemotherapy constitute core components within the treatment standard for advanced HNSCC. However, the high recurrence rate and poor overall survival demand the development of a more effective therapeutic strategy. Evidence indicates that although tumors are effectively debulked by conventional therapies, the distinctively resistant cancer stem cells are not eradicated. In fact, platinum-based agents and cytotoxic chemotherapies have been demonstrated to increase the CSC fraction in tumors [13, 14, 35]. However, using only a CSC-targeted approach as a novel treatment strategy would result in remnant bulk tumor cells with residual growth potential. In an attempt to achieve optimal therapeutic outcomes, the CSC hypothesis explains that a combination therapy approach is required to target both CSC and bulk tumor cells, which could effectively prevent disease progression. Our data illustrate this effect in that Tocilizumab alone successfully decreases self-renewal within tumors, but is not as potent in reducing tumor growth as in combination with Cisplatin.

Targeting signaling conduits that compose critical roles in the maintenance of CSC might sensitize them to standard platinum-based chemotherapy and provide better treatment outcomes. Our lab has extensively described the role of endothelial cell-secreted IL-6 inside the perivascular niche in supporting the maintenance of the CSC pool and their invasive properties [24, 25]. Our results further our previous observations that IL-6 augments Cisplatin-induced cancer cell stemness, implicating Cisplatin and IL-6R signaling as mediators of phenotypic changes in HNSCC tumors that result in enhanced stemness [16]. Here we showcase a pioneering potential therapeutic strategy to suppress these adverse effects of Cisplatin treatment, which result in an increase in CSC. We have shown here that IL-6R inhibition using Tocilizumab can resolutely overcome CSC induction by Cisplatin and suppress the growth of Cisplatin-resistant tumors.

Further, we showed that both genetic and pharmacologic inhibition of IL-6R signaling could suppress Cisplatin-mediated induction of the CSC fraction, Bmi-1 expression, and self-renewal of HNSCC cells. Interestingly, we observed that IL-6R inhibition with Tocilizumab decreases the expression of IL-6R and downstream STAT3 signaling, which has recently also been shown in another study in triple‐negative breast cancer cells [36]. This might be explained by either internalization of the receptor via endocytosis and subsequent degradation, or by its shedding to increase levels of soluble IL-6R [37]. We have also made the rather surprising observation that Tocilizumab inhibits gp130 expression, even in the presence of Cisplatin. Collectively, our data suggest that IL-6R signaling plays an essential role in resistance to Cisplatin, which is frequently observed in patients with HNSCC. This observation, together with the fact that developing and obtaining FDA approval for a new drug proves highly difficult and costly, unveils the re-purposing of Tocilizumab as a highly attractive adjunct therapy with Cisplatin in a novel treatment strategy for HNSCC.

We observed here that combination therapy with Tocilizumab and Cisplatin is effective in inhibiting cancer stemness and tumor growth. It is well known that Cisplatin targets actively dividing, rapidly proliferating cells and triggers apoptosis. CSC resistance to conventional chemotherapeutics might be explicated by the fact that these cells are slow cycling. It is also known that Cisplatin does not kill CSCs, promotes CSC self-renewal (Bmi-1 induction), and that Cisplatin-resistant cells express elevated levels of stemness markers [13, 16]. These observations suggest that Cisplatin-mediated increase in CSC fraction results from preferential eradication of non-CSC, while simultaneously promoting CSC accumulation through self-renewal. The combined effect of Cisplatin and Tocilizumab observed in many of our data sets perhaps could be explained similarly. Although Tocilizumab inhibits cancer stemness, Cisplatin targets primarily rapidly proliferative bulk tumor cells but does not kill CSCs. As such, we propose here that the combination of both therapies is effective at ablating CSCs, while inhibiting tumor growth. Indeed, we observed that IL-6R inhibition with Tocilizumab can potently suppress Cisplatin-mediated induction of Bmi-1 expression and Cisplatin-mediated increase in CSC fraction. Interestingly, others have shown that Bmi-1^+^ cells represent a subset of CSCs that might be responsible for therapeutic resistance and tumor recurrence [18]. These observations underline the importance of elucidating the signaling mechanisms governing CSC self-renewal and stemness, and provide a potential mechanism by which CSC can be re-programmed and re-sensitized to conventional chemotherapies. Further investigation of mechanisms mediating this shift in the CSC fraction may explore the role of IL-6R signaling in CSC plasticity, as well as CSC symmetric vs. asymmetric cell division fates.

Bmi-1 is a principal controller of stem cell self-renewal [38–41]. IL-6/STAT3 signaling has been revealed to choreograph epithelial-mesenchymal transition through activation of Bmi-1, a process known to confer tumors with self-renewal and migratory abilities [42, 43]. Bmi-1 is uniquely expressed in head and neck CSC (as opposed to bulk tumor cells), and we showed that this effect is enhanced by IL-6 signaling [24]. It has been recently shown that direct inhibition of Bmi-1 abrogates CSC function and sensitizes cells to Cisplatin therapy in HNSCC [18]. Here we present data in support of the function of the IL-6R pathway in the modulation of Bmi-1 expression in HNSCC. Through both genetic and pharmacologic approaches, we demonstrated a compelling link between IL-6R signaling and Bmi-1 expression. The clinical importance of Bmi-1 in HNSCC was demonstrated through retrospective analysis of 216 patient samples that displayed a correlation among Bmi-1 levels and clinical outcomes. Interestingly, we found that Bmi-1 expression significantly correlated with recurrence-free patient survival time, which can be clarified by the CSC hypothesis. Although the small CSC fraction within a tumor may not noticeably contribute to overall tumor growth, it is resistant to radiation and conventional chemotherapy, and promotes tumor recurrence. Using Bmi-1 as a putative prognostic marker may enable risk assessment for recurrence in patients with HNSCC.

Collectively, these data provide preclinical evidence for an innovative mechanism-based treatment strategy that is based on targeted ablation of CSC with Tocilizumab in combination with Cisplatin to debulk the tumor. This new combination therapy has the potential to improve the survival and standard of health for HNSCC patients.

## Materials and methods

### Cisplatin-resistant cell lines and cell culture

Human HNSCC cell lines UM-SCC-1, UM-SCC-22A, and UM-SCC-22B (from T. Carey, University of Michigan) cultured in Dulbecco’s modified Eagle medium (Invitrogen, Carlsbad, CA, USA) with 10% fetal bovine serum (Atlanta Biologicals, Flowery Branch, GA, USA), 1% l-Glutamine (Sigma, Burlington, MA, USA), and 1% Antibiotic-Antimycotic solution (Sigma). The cell lines’ origin, confirmation of identity, and authentication by short tandem repeat profiling are described elsewhere [44] and tested negative for mycoplasma (Mycoplasma Detection Kit, Invitrogen). Cisplatin-resistant cell line variants were produced from UM-SCC-1, UM-SCC-22A, and UM-SCC-22B cells, as described previously [16, 45]. Four Cisplatin-resistant variants were generated for each parent cell line, e.g.: UM-SCC-1Cis1 (UM-SCC-1 resistant to 1 µM Cisplatin), UM-SCC-1Cis4 (UM-SCC-1 resistant to 4 µM Cisplatin), UM-SCC-1Cis6 (UM-SCC-1 resistant to 6 µM Cisplatin), and UM-SCC-1Cis12 (UM-SCC-1 resistant to 12 µM Cisplatin). Cisplatin (Sigma) treatment was removed from the passage before the experiments were performed, waiting at least 2 days until cells were utilized for experiments.

### HNSCC PDX mouse model

HNSCC tumor fragments were implanted into the dorsal subcutaneous space of immunodeficient adult male mice (CB17 SCID, Charles River, Wilmington, MA, USA), as previously described and characterized [29]. Once tumors grew to an average volume of 450 mm^3^, mice were randomly assigned to treatment groups (*n* = 5–8): 5 mg/kg Cisplatin (Sigma), 10 mg/kg Tocilizumab (Genentech); 10 mg/kg Tocilizumab + 5 mg/kg Cisplatin, or no treatment. Treatment was administered weekly intraperitoneally for 2 weeks. Mice were killed and tumors retrieved 2 days after the end of treatment. Tumor measurements were taken two to three times per week and volumes were calculated via the equation *V* = length × width^2^/2. Notably, PDX tumors of in vivo passage five or below were used in this manuscript. All procedures and treatments were conducted under protocols reviewed and approved by the University of Michigan UCUCA (PRO00009324).

### HNSCC subcutaneous scaffold xenograft mouse model

HNSCC subcutaneous xenograft tumors were generated as previously described [46] without the inclusion of HDMEC (Lonza, Walkersville, MD, USA) cells. Briefly, 1 × 10^5^ tumor cells (UM-SCC-22B and UM-SCC-22BCis6) were seeded with a cell growth media and Matrigel (Corning, Corning, NY, USA) mixture in poly-(l)-lactic acid (Sigma) biodegradable scaffoldings and subsequently implanted into the dorsal subcutaneous space of SCID mice (CB17, Charles River). For long-term treatments, mice were randomly assigned to treatment groups and dosages as described above once tumors reached an average volume of 250 mm^3^ (*n* = 6).

### Flow cytometry

Tumors were resected from mice, dissociated by collagenase and hyaluronidase (StemCell Technologies, Vancouver, BC, Canada), incubated in ACK red blood cell lysis buffer (Invitrogen), and filtered through a sterile 40 µm cell strainer. ALDH enzymatic activity was stained using Aldefluor Kit (StemCell Technologies) or AldeRed ALDH Detection Assay (MilliporeSigma, Burlington, MA, USA). Briefly, 1 × 10^6^ cells were incubated with activated ALDH substrate or the equivalent volume of ALDH inhibitor diethyl aminobenzaldehyde (DEAB). DEAB controls were included for all treatment conditions. Cells were rinsed with PBS and stained for CD44 with either CD44-PE or CD44-APC (R&D Systems, Minneapolis, MN, USA) for 15 min at 4 °C. Human cells were identified by anti-HLA-ABC (PE; BD Pharmingen, NJ, USA). Viable cells were stained with DAPI (Molecular Probes, Eugene, OR, USA). For cell sorting, ALDH^high^CD44^high^ CSC population was sorted against the remaining bulk tumor cells (i.e., ALDH^high^CD44^low^, ALDH^low^CD44^high^, and ALDH^low^CD44^low^). All flow cytometry analyses were conducted in a BD LSRFortessa flow cytometer (BD Biosciences). Results were analyzed with FlowJo software (LLC; Ashland, OR, USA) in triplicate wells per condition.

### Orosphere assay

HNSCC cells were grown in ULA culture ware (Corning) as previously described [28]. Twelve thousand cells/well were passed through a single-cell strainer and were seeded in six-well ULA plates. Twenty-four hours later, cells were treated with 1 µM Cisplatin (Sigma) and/or 0.1 µM Tocilizumab (Genentech). Primary orospheres were cultured by gradually adding media over time, while maintaining the drug concentration constant. Orospheres were dissociated on day 10 with Accutase (StemCell Technologies), passed through a sterile single-cell strainer, and re-plated at the same cell density to generate secondary orospheres. Secondary orospheres were not further treated and again cultured for 10 days. Orospheres were stated as non-adherent spheres containing ≥25 cells, as observed at high power magnification (×100 to ×200). Results are representative of that at minimum two independent experiments, all performed in triplicate experimental conditions. Coefficient of drug interaction (CDI) was calculated to analyze the effect of the combination therapy, as follows: CDI = *AB*/(*A* × *B*). *AB* represents the ratio of the combination therapy to control, whereas *A* or *B* represent the ratio of the individual treatments to control. CDI < 1 specifies synergism (for CDI < 0.7 significantly synergistic effect), CDI = 1 specifies an additive effect, and CDI > 1 specifies antagonism.

### Pluripotent stem cell array

The proteome profiler human pluripotent stem cell array kit (R&D Systems) was used to evaluate expression of stem cell markers, according to the manufacturer’s instructions. Briefly, UM-SCC-1, UM-SCC-22A, or UM-SCC-22B cells were plated, serum-starved overnight, and treated with 0–1 µM Cisplatin (Sigma) and/or 0–0.1 µM Tocilizumab (Genentech) for 24 h. Lysates were extracted and incubated with the antibody-spotted array following the manufacturer’s instructions. Biotinylated detection antibodies and streptavidin-horseradish peroxidase reagents enabled subsequent signal detection by chemiluminescence. The stem cell array was exposed on film and relative integrated densities of each dot were quantified using ImageJ software (NIH, Bethesda, MA, USA).

### Statistical analysis

Statistical analysis was achieved using GraphPad Prism (GraphPad, San Diego, CA, USA). One-way analysis of variance followed by appropriate post hoc tests (Tukey’s test) was used to analyze comparisons between two or more groups. Two-tailed Student’s *t*-test followed by appropriate post hoc tests (Mann–Whitney *U*-test) was used to compare two groups. Kaplan–Meier graphs were evaluated using the Gehan Breslow–Wilcoxon test. Statistical significance was defined at *p* < 0.05 throughout the manuscript. Intensity scores for individual TMA cores were determined and averaged within patients across multiple cores by senior oral pathologists blinded to patient information. Comparisons between levels of a clinical factor were tested for significance by nonparametric Kruskal–Wallis test. Univariate and multivariable Cox regression models (adjusted for age, clinical stage, disease site, comorbidities, HPV status, and smoking) tested association with overall patient survival and recurrence-free time. For an illustration of adjusted analysis, adjusted survivor functions for intensity tertiles were plotted from the multivariable model. Statistical analysis of TMA data was performed in SAS v9.4. Sample sizes for in vitro and in vivo studies were determined by power calculations using data published in previous publications (or pilot tests) as reference. The variance between groups was relatively similar in the studies included here.

## Supplementary information

Supplementary methods and figures

## Data Availability

The data sets used and/or analyzed during the current study are available from the corresponding authors on reasonable request.
