# Supplementary material for: The IL-6R and Bmi-1 axis controls self-renewal and chemoresistance of head and neck cancer stem cells
Source: Cell Death Dis. 2021 Oct 23;12(11):988. doi: 10.1038/s41419-021-04268-5 (PMC8542035; doi:10.1038/s41419-021-04268-5)
Supplement: Supplementary file 1 — Supplementary methods and figures [file 41419_2021_4268_MOESM1_ESM.pdf]

## Supplementary Figure Legends

**Supplementary Figure S1. Flow cytometry gating and orosphere size measurements for CSC analysis of HNSCC cells stably transduced with shRNA-IL-6R. (A)** Flow cytometry plots depicting DEAB/IgG controls for Aldefluor and CD44, respectively. One experimental replicate per group is shown to demonstrate gate setting strategy. $ALDH^{high}CD44^{high}$ cells were identified based on these gates. **(B)** Bar graphs depicting the size of orospheres generated by HNSCC cells stably transduced with shRNA-IL-6R or scrambled vector control. Cells were treated with rhIL-6 (0 or 20 ng/ml). Different low case letters depict statistical significance at $p<0.05$.

**Supplementary Figure S2. Therapeutic effect of Tocilizumab and/or Cisplatin in a xenograft model of HNSCC. (A)** Schematic diagram depicting study design. Xenograft tumors were generated upon subcutaneous transplantation of UM-SCC-22B cells. Treatment was delivered for two weeks (three doses total), receiving either vehicle control, Cisplatin (5 mg/kg, I.P.) and/or Tocilizumab (10 mg/kg, I.P.). Following this initial treatment, mice that had incorporated Tocilizumab in their treatment plan continued receiving weekly maintenance injections of Tocilizumab (10 mg/kg). Mice were euthanized three weeks post main treatment end, or when they reached maximum tumor volume (2,000 $mm^3$). **(B)** Graphs depicting tumor volumes at treatment start. **(C)** Graphs depicting tumor volume at the end of experiment ($V_0$) normalized against tumor volume at treatment start ($V_i$). **(D)** Line graph depicting mean tumor volume over time after main treatment end, when only Tocilizumab maintenance treatment was administered to the corresponding groups. Tumor measurements were taken 3 times per week until study endpoints. **(E)** Simple linear regression model of mean tumor volumes over the duration of the experiment. $p=0.0356$ for comparison of the combination to control group.

**Supplementary Figure S3. Effect of Tocilizumab and/or Cisplatin on cancer stemness. (A)** Representative immunofluorescence images of UM-SCC-22A cells grown in chamber slides and stained for ALDH (green), Bmi-1 (red), and DAPI (blue) were captured at 200x. **(B)** Table depicting

the raw data from the stem cell marker protein array analysis of UM-SCC-1, UM-SCC-22A, and UM-SCC-22B cells treated with vehicle, Cisplatin (1 µM) and/or Tocilizumab (0.1 µM).

**Supplementary Figure S4. Tocilizumab inhibits orosphere growth and Bmi-1 expression** **(A)** Graph depicting orosphere growth over time after treatment with vehicle control, Cisplatin (1 µM) and/or Tocilizumab (0.1 µM). Cells were treated the day after plating in ultra-low attachment conditions. **(B)** Bar graphs depicting the size of primary orospheres after treatment with vehicle control, Cisplatin (1 µM) and/or Tocilizumab (0.1 µM). Different low case letters depict statistical significance at $p<0.05$. **(C)** Bar graphs depicting the size of secondary orospheres after treatment with vehicle control, Cisplatin (1 µM) and/or Tocilizumab (0.1 µM). Different low case letters depict statistical significance at $p<0.05$. **(D)** Western blot analysis of UM-SCC-1 and UM-SCC-22B cells treated with vehicle, rhIL-6 (0-20 ng/ml), Cisplatin (0-2 µM) and/or Tocilizumab (0 or 0.1 µM) for 24 hours. **(E)** Western blot analysis of UM-SCC-1 cells treated with vehicle, rhIL-6 (20 ng/ml), Cisplatin (1 µM) and/or Tocilizumab (0.1 µM) for 24 hours. Pre-treatment with Tocilizumab was delivered 1 hour prior to subsequent combination treatment. **(F)** Bar graph depicting the quantification of Bmi-1 protein expression normalized to GAPDH, from the Western blot depicted in panel S4E.

**Supplementary Figure S5. Flow cytometry gating for CSC analysis of Cisplatin-resistant HNSCC cells.** **(A)** Flow cytometry gating strategies depicting DEAB/IgG controls for Aldefluor and CD44, respectively. One experimental replicate per group is shown to demonstrate gate setting strategy. $ALDH^{high}CD44^{high}$ cells were identified based on these gates.

**Supplementary Figure S6. Tocilizumab decreases size of orospheres in Cisplatin-resistant HNSCC cell line variants.** **(A)** Representative images (40x) of naïve and Cisplatin-resistant UM-SCC-22A and UM-SCC-22B primary orospheres on day 8 after treatment with vehicle, Cisplatin (1 µM) and/or Tocilizumab (0.1 µM). Cells were treated the day after plating in ultra0low attachment conditions. Inserts are at 100x magnification. **(B)** Bar graphs depicting the size of orospheres generated from Cisplatin resistant cell line variants treated with vehicle, Cisplatin (1

μM) and/or Tocilizumab (0.1 μM). Different low case letters depict statistical significance at $p<0.05$. **(C)** Coefficient of drug interaction (CDI) for the combination effect of Cisplatin and Tocilizumab in primary orosphere formation, where CDI<1, =1, and >1 indicate synergism, additive effect, and antagonism (respectively).

**Supplementary Figure S7. Effect of Tocilizumab and/or Cisplatin in a Cisplatin-resistant xenograft model.**

(**A,B**) Western blots depicting the impact of Cisplatin and/or Tocilizumab on the expression of STAT3 and Bmi-1 in lysates prepared from xenograft tumors generated with UM-SCC-22BCis0 (A) or UM-SCC-22BCis6 (B) cells. Lysates were prepared from whole tumors upon dissociation (n=6 per experimental condition). (**C-F**) Graphs depicting the quantification of STAT3 (C,E) and Bmi-1 (D,F) protein expression normalized to GAPDH in tumors generated with UM-SCC-22BCis0 (C,D) or UM-SCC-22BCis6 (E,F) cells. Different low case letters depict statistical significance at $p<0.05$. **(G)** Graphs depicting tumor volumes at start of treatment.

## Supplementary Materials & Methods

### IL-6R gene silencing

HEK293T cells were used to produce lentiviral particles by co-transfecting packaging vectors pMD2.G and psPAX2 with either shRNA-control or shRNA-IL6R constructs on a pGIPZ backbone (University of Michigan Vector Core) using the calcium phosphate method. The supernatant was collected, and UM-SCC cells were infected overnight with the supernatant in the presence of 4 µg/ml polybrene (Sigma). Successfully infected cells were selected with 1 µg/ml puromycin (Invivogen, San Diego, CA).

### Western blot

Cells were plated, serum-starved overnight, and treated with vehicle, 0-1 µM Cisplatin (Cisplatin) and/or 0.1 µM Tocilizumab (Genentech). Alternatively, cells were pre-incubated with 0.1 µM Tocilizumab for 1 hour and then treated with 1 µM Cisplatin and/or 0-20 ng/ml rhIL-6 for 30 min or 24 hours. HNSCC cells and tumor tissues were lysed in NP-40 lysis buffer and loaded onto 9% SDS-PAGE gels. Membranes were blocked with 5% nonfat milk in TBST, then incubated with the following primary antibodies overnight at 4°C: p-STAT3, STAT3, Bmi-1, gp130, OCT4, Nanog (Cell Signaling, Danvers, MA, USA), GAPDH (Fisher Scientific, Hampton, NJ, USA), IL-6Rα (Santa Cruz, Dallas, TX, USA). Secondary anti-mouse or anti-rabbit antibodies conjugated with HRP (Jackson Laboratories, Bar Harbor, ME, USA) were used, and proteins were visualized by SuperSignal West Pico chemiluminescent substrate (Thermo Fisher Scientific, Waltham, MA, USA).

### Histological staining and analyses

Formalin-fixed, paraffin-embedded tissue sections were deparaffinized with xylene and rehydrated with graded ethanol. Orospheres were cryosectioned, and OCT compound was removed using PBS. HNSCC cells were plated in 4-well chamber slides, incubated overnight, and treated as described above. Chamber slide cultures were fixed in 10% buffered formalin. For immunohistochemical and immunofluorescence analyses, antigen retrieval was performed in

citrate buffer (Thermo Fisher Scientific) using a decloaking chamber following the manufacturer's instructions (Biocare Medical, Pacheco, CA, USA). Sections were incubated with 0.1% Triton X-100 (Fisher Scientific), followed by 3% hydrogen peroxide (Fisher Scientific), and Background Sniper (Biocare Medical). Orosphere sections were not incubated in hydrogen peroxide. Sections were exposed to primary antibodies at 4˚C overnight: anti-human ALDH1 (1:200; Rabbit; Abcam, Cambridge, UK), anti-human CD44 (1:800; Mouse; Cell Signaling), anti-human Bmi-1 (1:200; Rabbit; Cell Signaling). For immunoperoxidase staining, sections were incubated with MACH3 probe and MACH3 HRP polymer (Biocare Medical), and then DAB until the desired staining was reached. Sections were counterstained with hematoxylin (Vector Laboratories, Burlingame, CA, USA). For immunofluorescence, specimens were incubated in secondary antibodies labeled with either mouse or rabbit Alexafluor 488 or 594 (Invitrogen, Waltham, MA, USA). Specimens were mounted in Vectashield mounting medium for fluorescence with DAPI (Vector Laboratories). Fluorescence intensity was measured in randomly selected fields (at least 4 in triplicate experimental conditions) using ImageJ and analyzed in GraphPad Prism (GraphPad). Images were captured with a Nikon Eclipse E800 fluorescence microscope or a Nikon confocal microscope.

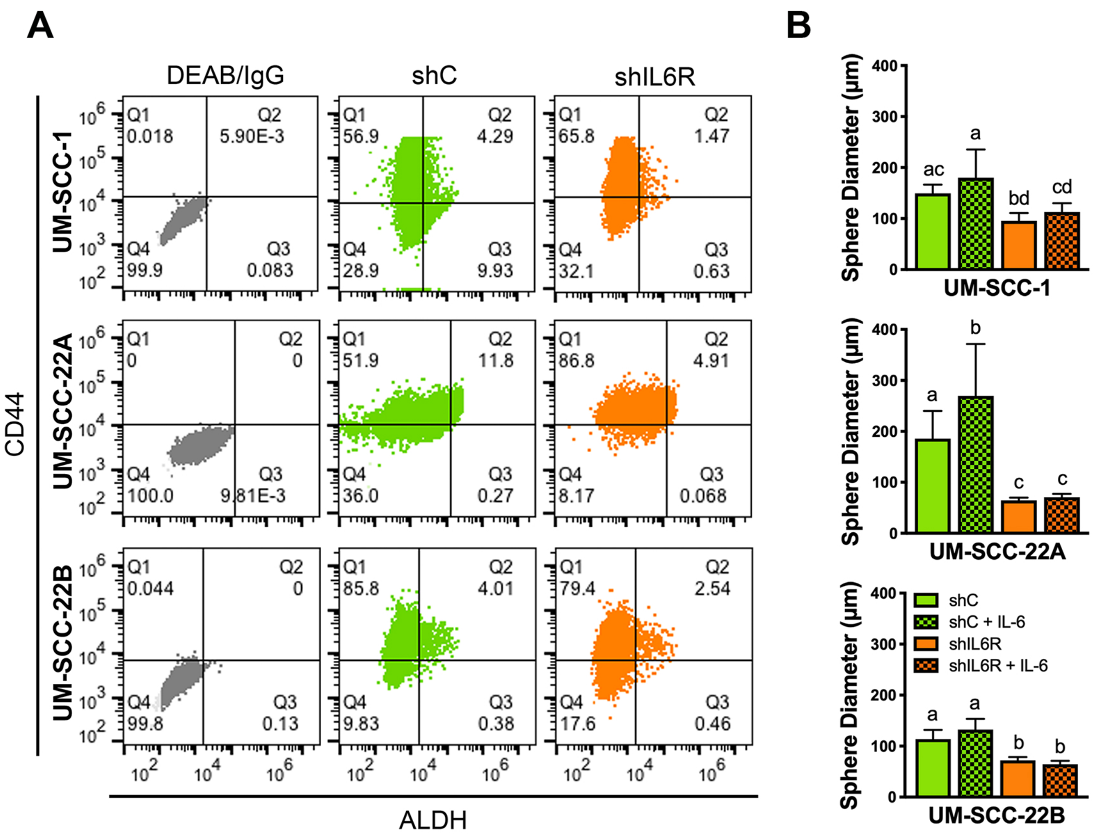


**Supplementary Figure S1** - Herzog et al

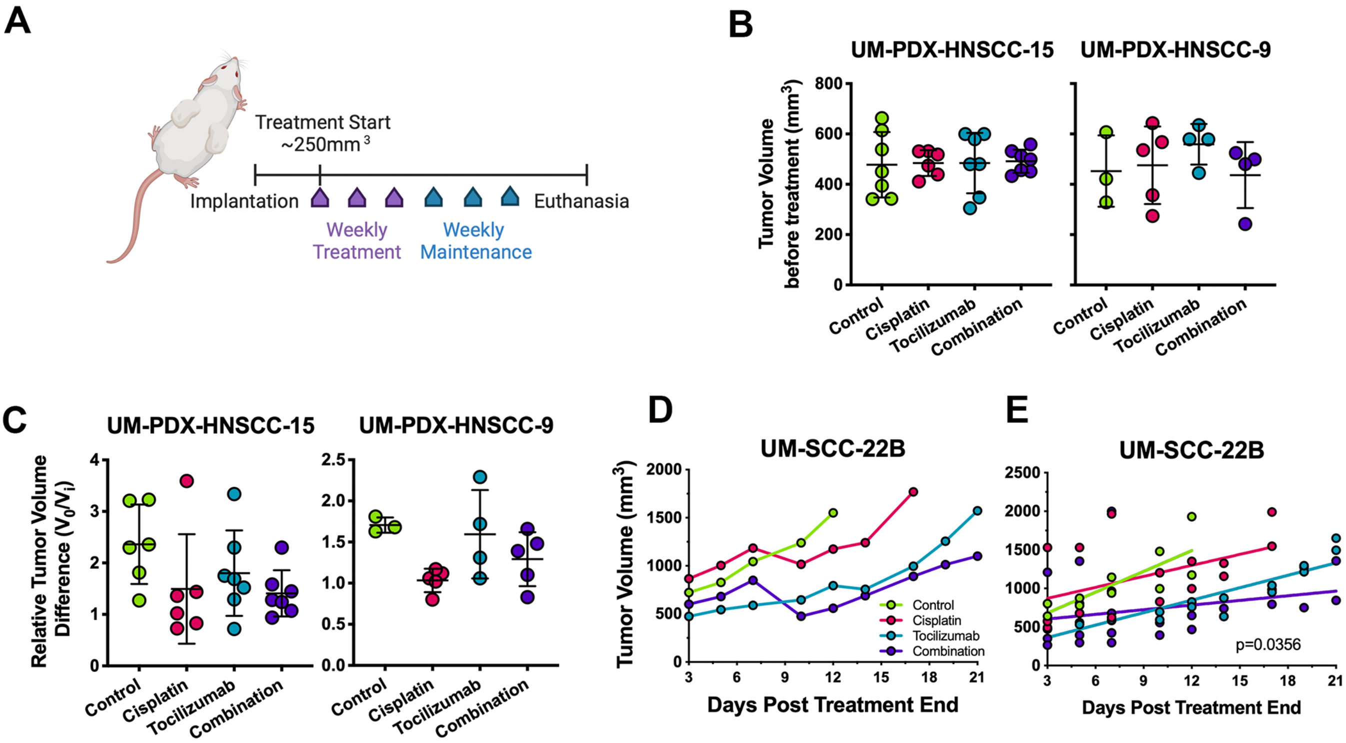


**Supplementary Figure S2** - Herzog

**A**

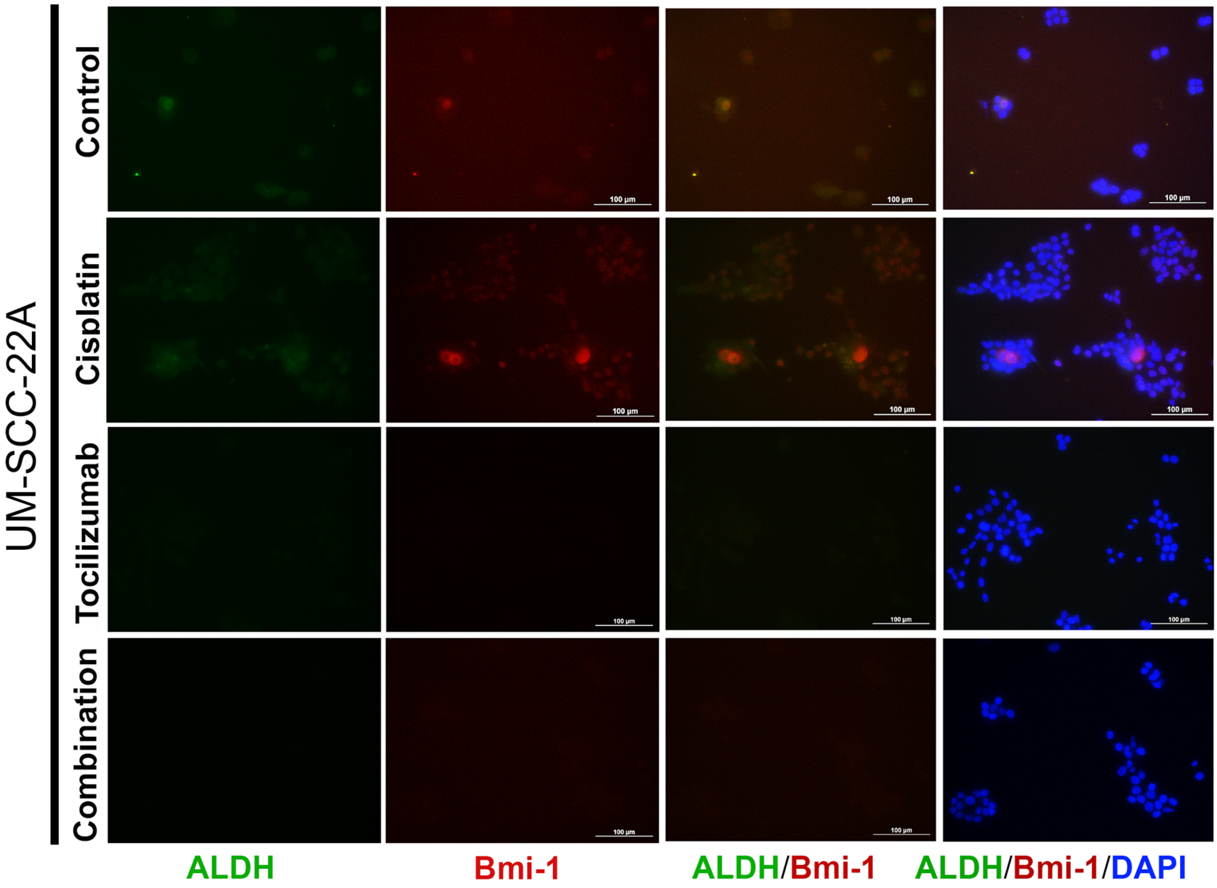


**B**

| | UM-SCC-1 | | | | UM-SCC-2A | | | | UM-SCC-22B | | | |
|---|---|---|---|---|---|---|---|---|---|---|---|---|
| | Control | Cis | Tcz | Cis + Tcz | Control | Cis | Tcz | Cis + Tcz | Control | Cis | Tcz | Cis + Tcz |
| **Oct3/4** | 0.088 | 0.078 | 0.071 | 0.023 | 0.134 | 0.135 | 0.121 | 0.089 | 0.118 | 0.082 | 0.053 | 0.053 |
| **Nanog** | 0.097 | 0.057 | 0.056 | 0.021 | 0.094 | 0.229 | 0.142 | 0.138 | 0.208 | 0.164 | 0.150 | 0.078 |
| **Sox2** | 0.117 | 0.075 | 0.082 | 0.019 | 0.285 | 0.349 | 0.369 | 0.239 | 0.216 | 0.147 | 0.162 | 0.082 |
| **IPF1** | 0.221 | 0.144 | 0.104 | 0.027 | 0.338 | 0.435 | 0.444 | 0.384 | 0.529 | 0.446 | 0.360 | 0.295 |
| **Sox17** | 0.086 | 0.070 | 0.047 | 0.021 | 0.098 | 0.179 | 0.159 | 0.113 | 0.156 | 0.120 | 0.065 | 0.069 |
| **Otx2** | 0.121 | 0.076 | 0.066 | 0.021 | 0.098 | 0.179 | 0.159 | 0.113 | 0.223 | 0.205 | 0.171 | 0.105 |

**Supplementary Figure S3** - Herzog et al

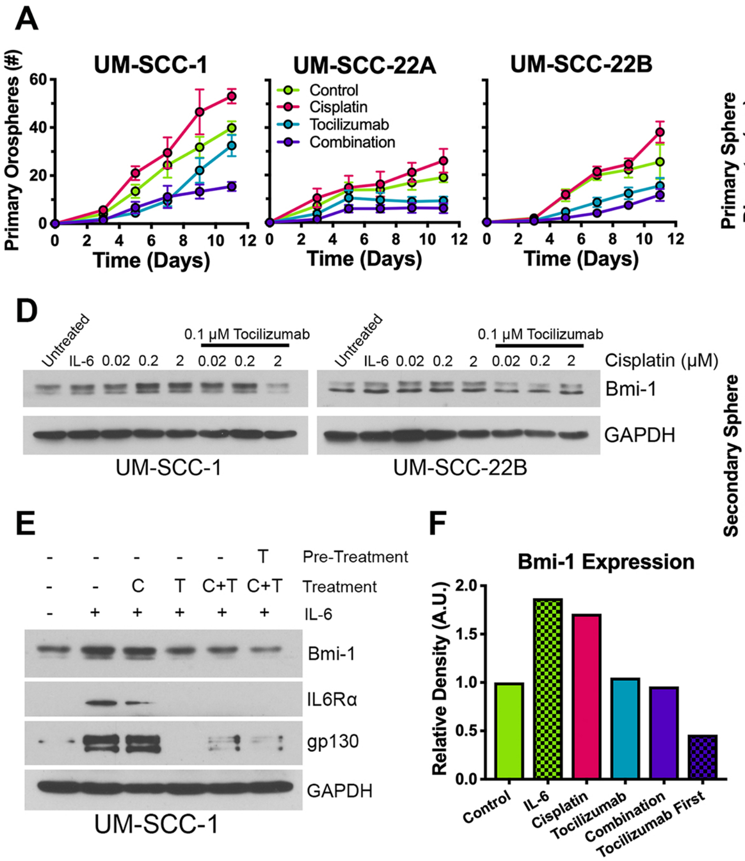


**Supplementary Figure S4** - Herzog et al

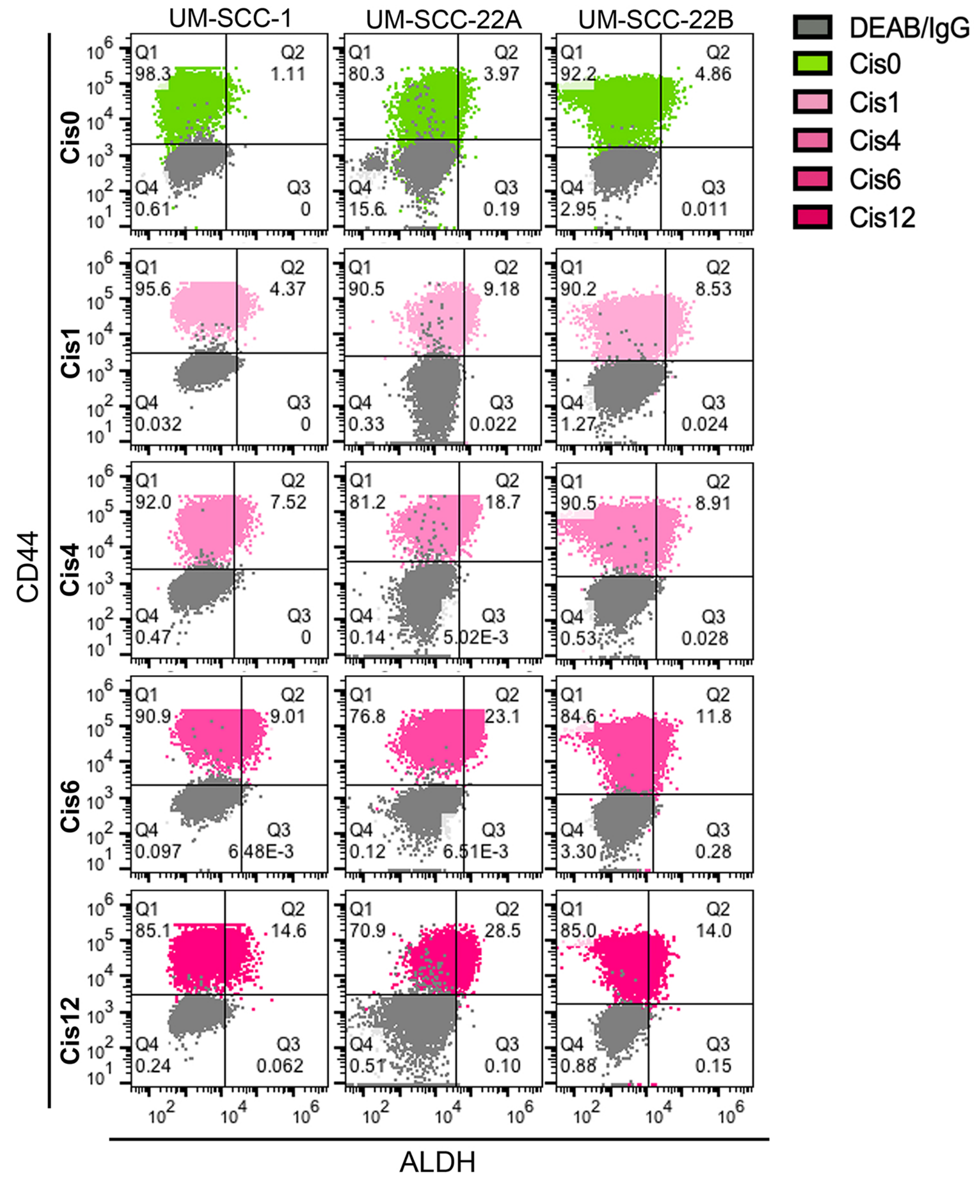


**Supplementary Figure S5** - Herzog et al

A

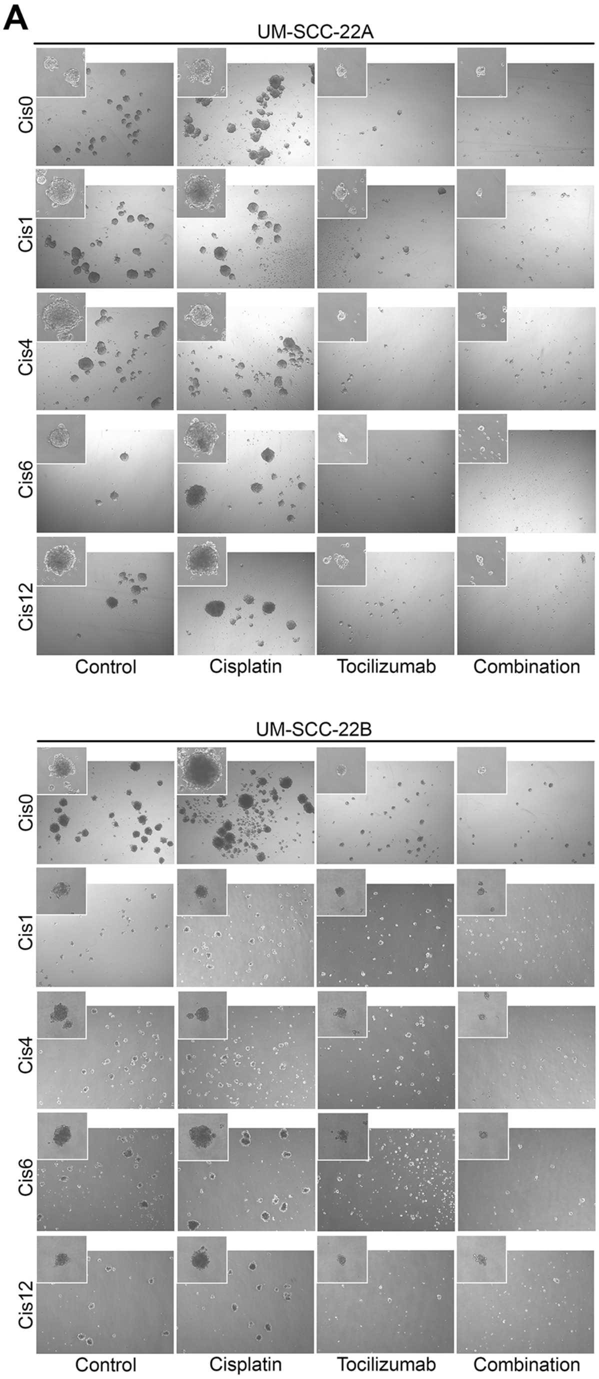


B

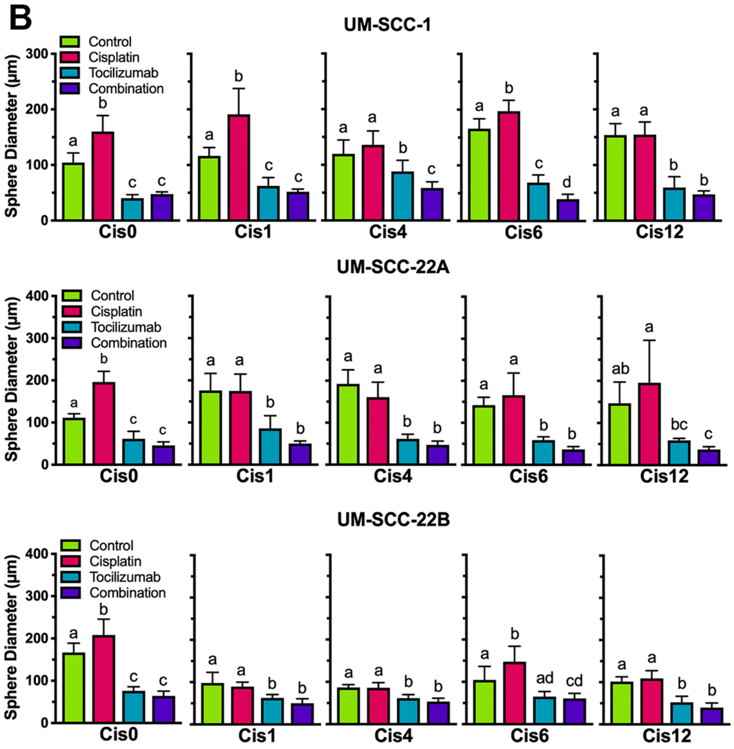


C

| | UM-SCC-1 | UM-SCC-22A | UM-SCC-22B |
|---|---|---|---|
| **Cis0** | 0.268 | 0.111 | 0.396 |
| **Cis1** | 0.388 | 0.198 | 0.311 |
| **Cis4** | 0.059 | 0.164 | 0.472 |
| **Cis6** | 0.751 | 0.160 | 0.515 |
| **Cis12** | 0.133 | 0.104 | 0.202 |

**Supplementary Figure S6** - Herzog et al

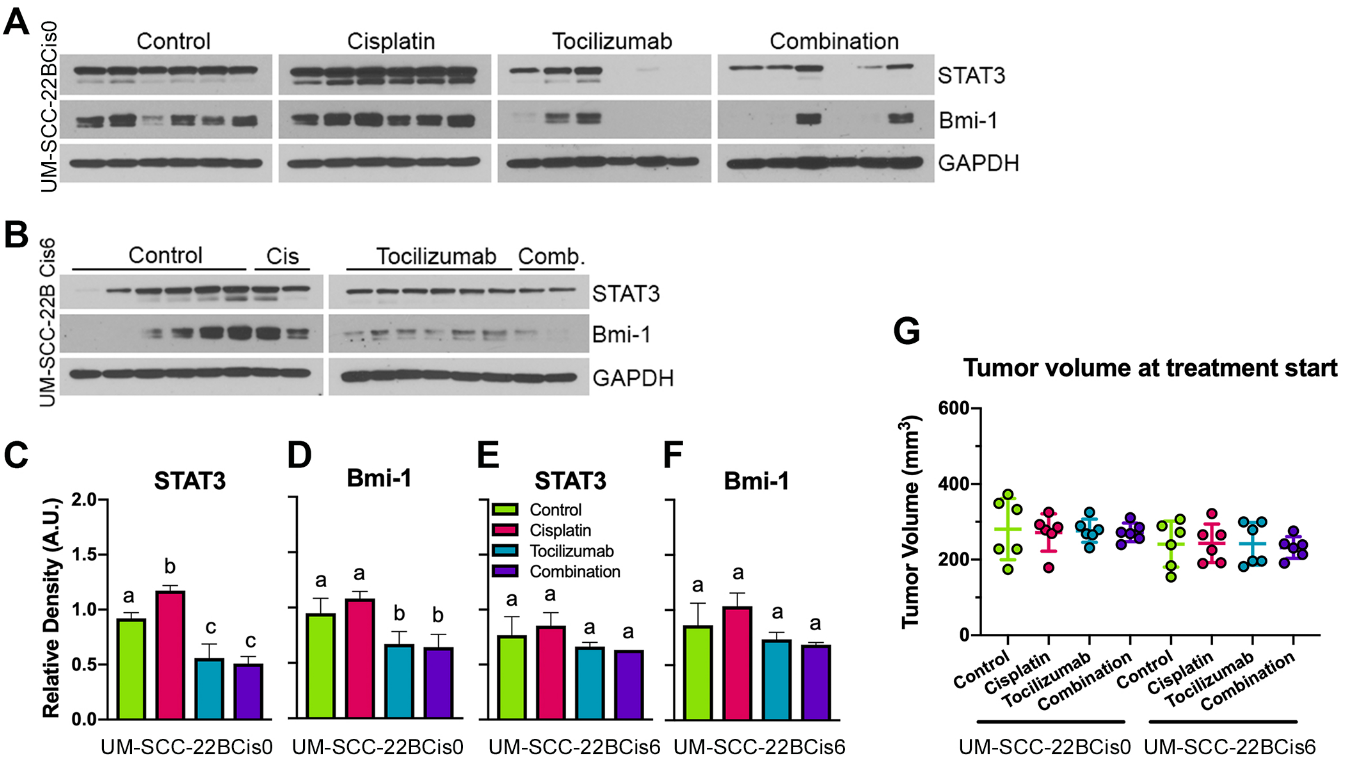


**Supplementary Figure S7** - Herzog et al
